# Combined assessment of inflammation and food intake contributes to prognostic stratification of gastric cancer

**DOI:** 10.3389/fonc.2025.1669838

**Published:** 2025-10-23

**Authors:** Yixuan Wang, Xi Zhang, Chenan Liu, Jinyu Shi, Tong Liu, Yue Chen, Xin Zheng, Zhaoting Bu, Hanping Shi

**Affiliations:** ^1^ Department of Clinical Nutrition, Beijing Shijitan Hospital, Capital Medical University, Beijing, China; ^2^ Key Laboratory of Cancer Food for Special Medical Purposes (FSMP) for State Market Regulation, Beijing, China; ^3^ Department of Gastrointestinal Surgery, Beijing Shijitan Hospital, Capital Medical University, Beijing, China; ^4^ Department of Comprehensive Oncology, National Cancer Center/National Clinical Research Center for Cancer/Cancer Hospital, Chinese Academy of Medical Sciences and Peking Union Medical College, Beijing, China; ^5^ The Third Department of Breast Cancer, Tianjin Medical University Cancer Institute and Hospital, Tianjin, China

**Keywords:** gastric cancer, lymphocyte to C-reactive protein ratio, food intake, nutrition, overall survival

## Abstract

**Background:**

Patients with gastric cancer (GC) present with chronic inflammation and malnutrition risk. Lymphocyte-to-C-reactive protein ratio (LCR) and food intake are promising indicators for predicting inflammatory and nutritional states.

**Methods:**

This multi-center cohort study included 763 patients with GC. Time-dependent receiver operating characteristic curves were generated to determine the prediction accuracy of 16 systemic inflammatory indicators. Association of the model constructed by LCR and food intake with overall survival (OS) were analyzed using the Kaplan–Meier method and Cox regression model.

**Results:**

In this analysis, patients with reduced food intake accounted for 60.4%. The area under the curve and C-index of LCR for all-cause mortality were higher than those of the other indicators in patients with GC and there was a significant inverse relationship between LCR and all-cause mortality (per SD increment HR: 0.79, 95% CI: 0.65–0.96; *P* = 0.016). Patients with reduced food intake had lower LCR than those patients without reduced food intake. Low LCR had combined effects with reduced food intake on unfavorable OS of patients with GC.

**Conclusions:**

Combined assessment of inflammation and food intake contributes to prognostic stratification of GC. Active therapeutic measures to reduce inflammation and increase nutrition may improve outcomes of affected patients.

## 1 Introduction

Gastric cancer (GC) is one of the most malignant cancers globally, ranking fifth in incidence and fourth in mortality (1). Increasing evidence shows malnutrition, immune escape, and systemic inflammatory response, are associated with poor outcomes in patients with GC (2–4). Tumor-related systemic inflammation plays a crucial role in the development and metastasis of tumor cells as it allows these cells to evade immune system recognition and subsequent destruction (5, 6). Systemic inflammatory response affects the cancer microenvironment, causing tumor cells to proliferate, metastasize, and weaken the response to anticancer drugs (7). Previous studies have shown that in addition to the well-established evidences supporting C-reactive protein (CRP) as a systemic inflammatory marker, lymphocytes could also be used to assess immune-nutrition status (8, 9). When combining lymphocytes and CRP, several scholars found that lymphocyte to C-reactive protein ratio (LCR) may be a more promising biomarker for reflection of the systemic immune-inflammation status in patients with malignancies.

A significant percentage of malnutrition at the time of diagnosis in patients with GC owing to the inherent characteristics and tumor factors, resulting in a deteriorating systemic metabolic response, or the inevitable progression of malnutrition owing to increasing chemoradiotherapy-induced toxicity, remains a clinical challenge for both patients and physicians (10–12). A recent study using the Global Leadership Initiative on Malnutrition (GLIM) reported that the prevalence of malnutrition risk in patients with GC was 53% (13). The mechanisms underlying the progression of malnutrition in patients with GC can be attributed to inadequate food intake and aberrant metabolism caused by varied degrees of systemic inflammation induced by cancer, treatment, or both (14, 15). Reduced food intake is a frequent finding in advanced malignant disease, especially GC. However, few studies have addressed the prognostic significance of the combined assessment of LCR and food intake in patients with GC.

We aimed to verify whether LCR is the best indicator for assessing the inflammation burden by comparing the prediction accuracy of 16 systemic inflammatory indicators including CRP, LCR, prognostic nutritional index (PNI), neutrophil to lymphocyte ratio (NLR), glucose to lymphocyte ratio (GLR), advanced lung cancer inflammation index (ALI), systemic immune inflammation index (SII), C-reactive protein to albumin ratio (CAR), controlling nutritional status score (CONUT), modified Glasgow prognostic score (mGPS), geriatric nutritional risk index (GNRI), modified geriatric nutritional risk index (mGNRI), albumin to globulin ratio(AGR), nutritional risk index(NRI), platelet to lymphocyte ratio (PLR), and lymphocyte to CRP ratio score (LCS), as well as to determine prognostic significance of the combined assessment of LCR and food intake in patients with GC.

## 2 Methods

### 2.1 Study population and design

This multicenter cohort study recruited 1543 patients aged 18–95 years, diagnosed with GC by pathology underwent routine examinations, who were enrolled at more than 40 clinical centers throughout China from April 2013 through December 2022. The specific inclusion criteria were as follows: (1) patients with gastric cancer; (2) length of hospital stay >48 h; and (3) diagnosis of solid tumors at any stage. Patients with incomplete clinical data or those lost to follow-up at the beginning and subsequent follow-up were excluded. Finally, 763 patients with GC were enrolled in this study. All participants signed informed consent forms prior to study entry (Registration number: ChiCTR1800020329; Date of trial registration: 24/12/2018). This study complied with the Declaration of Helsinki and was approved by the institutional ethics committees of Beijing Shijitan Hospital.

### 2.2 Data collection and variable definition

We collected information on the clinicopathological characteristics of all participants, including age, gender, alcohol consumption, smoking status, previous treatments (surgery, chemotherapy and radiotherapy), TNM stage, Karnofsky Performance Status (KPS) and laboratory routine blood tests. Blood samples from all patients were drawn within 48 hours after admission. Counts of neutrophils and lymphocytes, as well as the levels of CRP and albumin were recorded. These measurements were standardized to account for systematic differences in location and/or scale of measurements between laboratories. The TNM stage was classified following the guidelines by the eighth AJCC TNM staging system. Inflammatory burden assessments were performed using several parameters including LCR, PNI, CAR, CRP, ALI, mGNRI, LCS, NRI, GNRI, AGR, NLR, CONUT, GLR, PLR, SII, and mGPS. LCR was defined as the lymphocyte count divided by the CRP ratio. PNI was calculated using the formula PNI = serum albumin concentration (g/L) + 5×absolute lymphocyte count (10^9/L) (16). The calculation formulas for all indicators are shown in **Supplementary Table S1**. Patient-generated subjective nutrition assessment (PG-SGA), KPS and self-reported symptoms were also taken and recorded by trained staff at baseline. Reduced food intake was assessed by the PG-SGA scale and some simple questions (Supplementary Methods).

### 2.3 Outcome evaluation

All patients were regularly followed up by telephone or outpatient visits to collect information on clinical outcomes. Overall survival (OS) time was defined as the interval between the first assessment in the clinic until the date of death, date of withdrawal from the study, or the time of the last follow-up. The primary objective was to verify whether LCR is the best indicator for assessing the inflammation burden by comparing the prediction accuracy of 16 systemic inflammatory indicators including CRP, LCR, PNI, NLR, GLR, ALI, SII, CAR, CONUT, mGPS, GNRI, mGNRI, AGR, NRI, PLR, and LCS, as well as to determine prognostic significance of the combined assessment of LCR and food intake in patients with GC.

### 2.4 Statistical analysis

Descriptive statistics are presented as mean ± standard deviation (SD) for continuous variables and as numbers for categorical variables. The continuous and categorical variables were compared using the Student’s t-test and the χ2 test, respectively. The area under the curves (AUC) and C-index were calculated to determine the best indicator for assessing the inflammation burden. Restricted cubic spline regression was performed to evaluate the association between LCR and OS. Maximally selected rank statistics were used to calculate the optimal cut-off value for LCR (17). Depending on the calculated cut-off point, the patients were classified into high LCR, low LCR, Non-Reduced food intake, and Reduced food intake groups for subsequent analysis. Cox proportional hazard models were used to calculate hazard ratios (HRs) and 95% confidence intervals (CIs). Model A adjusted for age, gender, tumor stage, and BMI, while Model B adjusted for age, gender, smoking, drinking, tumor stage, BMI, KPS, PG-SGA, surgery, radiotherapy, and chemotherapy. Survival curves were plotted using the Kaplan–Meier method and the Log rank test. We also conducted a sensitivity analysis. Given that chronic inflammatory diseases may affect the results of this study, we excluded patients with known inflammatory bowel disease, chronic obstructive pulmonary disease, rheumatic diseases, and Alzheimer’s disease. All two-tailed statistical P values <0.05 were considered statistically different. All analyses were performed using R software, version 4.0.5.

## 3 Results

### 3.1 Patient characteristics

In this study, a total of 763 cases were enrolled in the cohort, the specific flow chart is shown in **Supplementary Figure S1**. Among the entire group, the age distribution was 59.12 ± 11.87 years, and 534 (70.0%) of the patients were male. Patients with reduced food intake accounted for 60.4% (**Supplementary Table S2**). Based on the LCR cut-off value of 6451.6 for OS, 234 (30.7%) and 529 (69.3%) patients were classified as having high and low LCR, respectively. The comparison of the patients’ demographic and clinicopathological characteristics between patients with and without reduced food intake, and the high and low LCR groups are presented in **Table 1**. There were significant associations between low LCR and old age, surgery, reduced food intake, higher TNM stages, PG-SGA, neutrophil count and lower KPS, serum albumin concentration, lymphocyte count, and PNI level. Reduced food intake was associated with higher TNM stages, PG-SGA and lower BMI, KPS, serum albumin concentration, lymphocyte count, LCR level, and PNI level.

**Table 1:** Characteristics of the study population with gastric cancer stratified by LCR and food intake.

Characteristics	High LCR	Low LCR	*P*	Non-reduced food intake	Reduced food intake	*P*
	(n=234)	(n=529)		(n=302)	(n=461)	
Age, mean (SD)	57.59 (12.46)	59.81 (11.54)	0.018	58.31 (11.84)	59.65 (11.87)	0.127
Gender, n (%)			0.185			0.406
Male	172 (73.5)	362 (68.4)		217 (71.9)	317 (68.8)	
Female	62 (26.7)	167 (31.5)		5 (28.1)	144 (31.2)	
BMI, mean (SD)	21.15 (3.04)	21.19 (3.18)	0.888	21.49 (3.18)	20.97 (3.09)	0.027
Surgery	115 (49.1)	316 (59.7)	0.008	169 (56.0)	262 (56.8)	0.870
Radiotherapy, n (%)			0.696			0.343
Yes	2 (0.9)	8 (1.5)		2 (0.7)	8 (1.7)	
No	232 (99.1)	521 (98.5)		300 (99.3)	453 (98.3)	
Chemotherapy, n (%)			0.079			0.945
Yes	103 (44.0)	271 (51.2)		149 (49.3)	225 (48.8)	
No	131 (56.0)	258 (48.8)		153 (50.7)	236 (51.2)	
TNM stages, n (%)			<0.001			<0.001
I	31 (13.2)	33 (6.2)		34 (11.3)	30 (6.5)	
II	65 (27.8)	84 (15.9)		74 (24.5)	75 (16.3)	
III	99 (42.3)	175 (33.1)		106 (35.1)	168 (36.4)	
IV	39 (16.7)	273 (44.8)		88 (29.1)	188 (40.8)	
PG-SGA, mean (SD)	6.52 (4.22)	8.29 (4.86)	<0.001	4.69 (3.52)	9.76 (4.35)	<0.001
KPS, mean (SD)	88.12 (8.63)	82.80 (11.97)	<0.001	87.09 (9.05)	82.69 (12.29)	<0.001
Albumin, g/L, mean (SD)	41.70 (4.81)	37.42 (5.09)	<0.001	40.23 (5.30)	37.75 (5.21)	<0.001
Neutrophil, 10^9^/L, mean (SD)	3.42 (1.56)	4.47 (4.02)	<0.001	3.87 (3.31)	4.33 (3.59)	0.074
Lymphocyte, 10^9^/L, mean (SD)	1.84 (0.65)	1.39 (0.58)	<0.001	1.59 (0.58)	1.49 (0.67)	0.029
Reduced food intake, n (%)			<0.001			<0.001
Yes	110 (47.0)	351 (66.4)		0(0)	461(100)	
No	124 (53.0)	178 (33.6)		302(100)	0(0)	
LCR, mean (SD)	24921.68 (27276.21)	2444.86 (1967.61)	<0.001	5168.15 (10218.79)	3125.00 (5738.10)	<0.001
PNI, mean (SD)	50.88 (5.77)	44.38 (6.19)	<0.001	48.19 (6.54)	45.19 (6.65)	<0.001

BMI, body mass index; PG-SGA, Patient-Generated Subjective Global Assessment; KPS, Karnofsky Performance Status; LCR, Lymphocyte-to-CRP ratio; PNI, Prognostic nutritional index.

### 3.2 LCR is the most accurate prognostic systemic inflammatory indicator for assessing the survival

**Figure 1:**
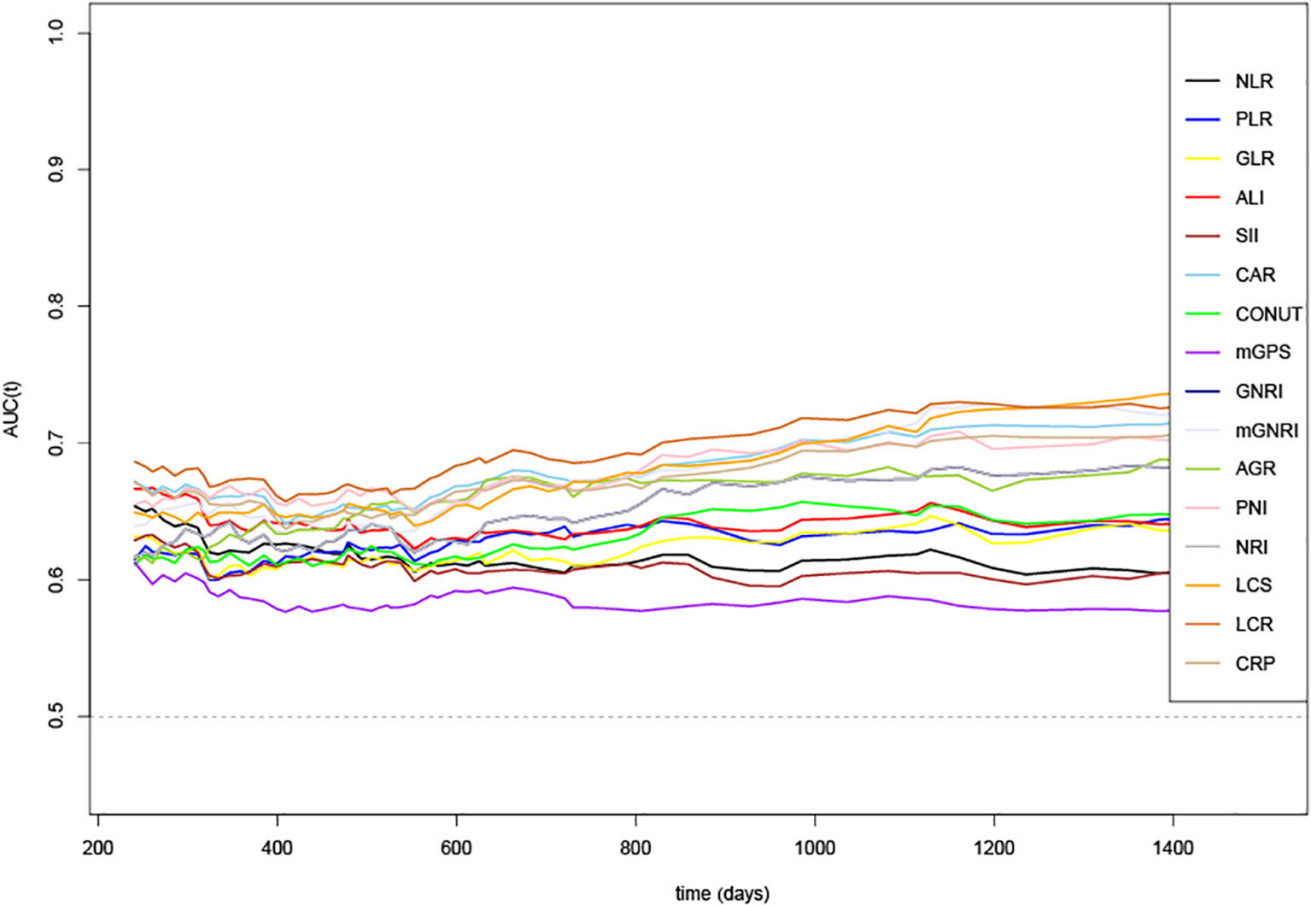
Time-dependent changes in the area under the curve (AUC) for overall survival of 16 systemic inflammatory indicators. LCR, Lymphocyte-to-CRP ratio; PNI, Prognostic nutritional index; NLR, Neutrophil-to-lymphocyte ratio; GLR, Glucose-to-lymphocyte ratio; ALI, Advanced lung cancer inflammation index; SII, Systemic immune inflammation index; CAR, C-reactive protein-to-albumin ratio; CONUT score, Controlling nutritional status score; mGPS, modified Glasgow prognostic score; GNRI, Geriatric nutritional risk index; mGNRI, modified Geriatric nutritional risk index; AGR, Albumin-to-globulin ratio; NRI, Nutritional risk index; PLR, Platelet-to-lymphocyte ratio; LCS, Lymphocyte-to-CRP ratio score; CRP, C-reactive protein.

Time-dependent changes in the AUCs of the 16 indicators for OS rate are shown in **Figure 1**. The AUC value of LCR was the highest than that of other indicators, indicating that LCR was the superior prognostic biomarker for predicting OS of patients with GC. Consistently, among the 16 systemic inflammatory indicators, the *C*-index values of all indicators were more than 0.5 in predicting OS, with LCR having the highest *C*-index value of 0.642(0.610, 0.674) (**Supplementary Table S3**).

### 3.3 Relationship between LCR levels or food intake and all-cause mortality

When analyzed as a continuous variable, restricted cubic splines showed a significant inverse relationship between LCR levels and all-cause mortality in patients with GC (per SD increase HR, 0.79; 95% CI: 0.65–0.96; *P* = 0.016) (**Supplementary Figure S2**, **Table 2**). We constructed different adjustment models to reduce clinical bias. Particularly, Model a was adjusted for age, gender, tumor stage and BMI; Model b was adjusted for age, gender, smoking, drinking, tumor stage, BMI, KPS, PG-SGA, surgery, radiotherapy, and chemotherapy. The risk of all-cause mortality was significantly higher in patients with low LCR than in those with high LCR (adjusted HR, 1.94; 95% CI: 1.46–2.58, *P* < 0.001). When LCR was classified into quartiles (Q1: >7511.68, Q2: 3973.29–7511.68, Q3: 1016.92–3973.29, Q4: < 1016.92), patients with LCR of Q2 (adjusted HR, 1.73; 95% CI: 1.2–2.49; *P* = 0.003), Q3 (adjusted HR, 1.92; 95% CI: 1.35–2.72; *P* < 0.001) and Q4 (adjusted HR, 2.36; 95% CI: 1.66–3.36; *P* < 0.001) were significantly correlated with worse prognosis compared with that of the Q1 group (**Table 2**). In addition, there was a significant increasing trend in the risk of all-cause mortality in patients with reduced food intake compared with individuals without reduced food intake, with an adjusted HR of 1.27 (95% CI: 0.97–1.68, *P* = 0.09).

**Table 2:** The association between LCR levels or Food intake and all-cause mortality in patients with gastric cancer.

Group	Crude model		Model a		Model b	
	HR 95%CI	*p*-value	HR 95%CI	*p*-value	HR 95%CI	*p*-value
LCR						
As continuous (per SD)	0.67 (0.55,0.82)	<0.001	0.78 (0.64,0.95)	0.012	0.79 (0.65,0.96)	0.016
By LCR cut-off						
High (6451.6~)	Ref.		Ref.		Ref.	
Low (~6451.6)	2.62 (2,3.43)	<0.001	1.94 (1.47,2.56)	<0.001	1.94 (1.46,2.58)	<0.001
Interquartile						
Q1 (7511.68~)	ref		ref		ref	
Q2 (3973.29~7511.68)	1.92 (1.34,2.74)	<0.001	1.68 (1.17,2.41)	0.005	1.73 (1.2,2.49)	0.003
Q3 (1016.92~3973.29)	2.65 (1.9,3.7)	<0.001	1.91 (1.36,2.68)	<0.001	1.92 (1.35,2.72)	<0.001
Q4 (~1016.92)	3.5 (2.51,4.88)	<0.001	2.41 (1.71,3.39)	<0.001	2.36 (1.66,3.36)	<0.001
P for trend		<0.001		<0.001		<0.001
Food intake status						
Non-Reduced food intake	ref		ref		ref	
Reduced food intake	1.73 (1.37,2.18)	<0.001	1.66 (1.30,2.13)	<0.001	1.27 (0.97,1.68)	0.09

Data were presented as hazard ratios (95% confidential intervals). LCR, Lymphocyte-to-CRP ratio.

Model a: adjusted for age, gender, tumor stage and BMI;

Model b: adjusted for age, gender, smoking, drinking, tumor stage, BMI, KPS, PG-SGA, surgery, radiotherapy, chemotherapy.

### 3.4 Association of the model constructed by LCR and food intake with all-cause mortality

In the analysis of the distribution of LCR levels in the reduced food intake population and non-reduced food intake population in the cohort, it was found that LCR levels were lower in patients with reduced food intake than those patients without reduced food intake (*P* < 0.001) (**Supplementary Figure S3**). **Table 3** shows the association of the model constructed by LCR and food intake with all-cause mortality. Compared with patients with high LCR and without reduced food intake, the patients with high LCR and reduced food intake, low LCR and without reduced food intake, and low LCR and reduced food intake were all positively correlated with worse prognosis (HR, 1.72; 95% CI: 1.04–2.84; HR, 2.54; 95% CI: 1.63–3.95; HR, 2.67; 95% CI: 1.72–4.15, respectively) after adjusting for the confounding factors.

**Table 3:** The association of the model constructed by LCR and Food intake with all-cause mortality in patients with gastric cancer.

Group	Crude model		Model a		Model b	
	HR 95%CI	*p*-value	HR 95%CI	*p*-value	HR 95%CI	*p*-value
High LCR, Non-Reduced food intake	ref		ref		ref	
High LCR, Reduced food intake	2.04 (1.26,3.31)	0.004	1.88 (1.16,3.04)	0.01	1.72 (1.04,2.84)	0.035
Low LCR, Non-Reduced food intake	3.1 (2.01,4.78)	<0.001	2.54 (1.64,3.93)	<0.001	2.54 (1.63,3.95)	<0.001
Low LCR, Reduced food intake	4.2 (2.8,6.28)	<0.001	2.79 (1.85,4.22)	<0.001	2.67 (1.72,4.15)	<0.001
P for trend		<0.001		<0.001		<0.001

Data were presented as hazard ratios (95% confidential intervals). LCR, Lymphocyte-to-CRP ratio.

Model a: adjusted for age, gender, tumor stage and BMI;

Model b: adjusted for age, gender, smoking, drinking, tumor stage, BMI, KPS, PG-SGA, surgery, radiotherapy, chemotherapy.

### 3.5 Survival outcomes of patients with GC

**Figure 2:**
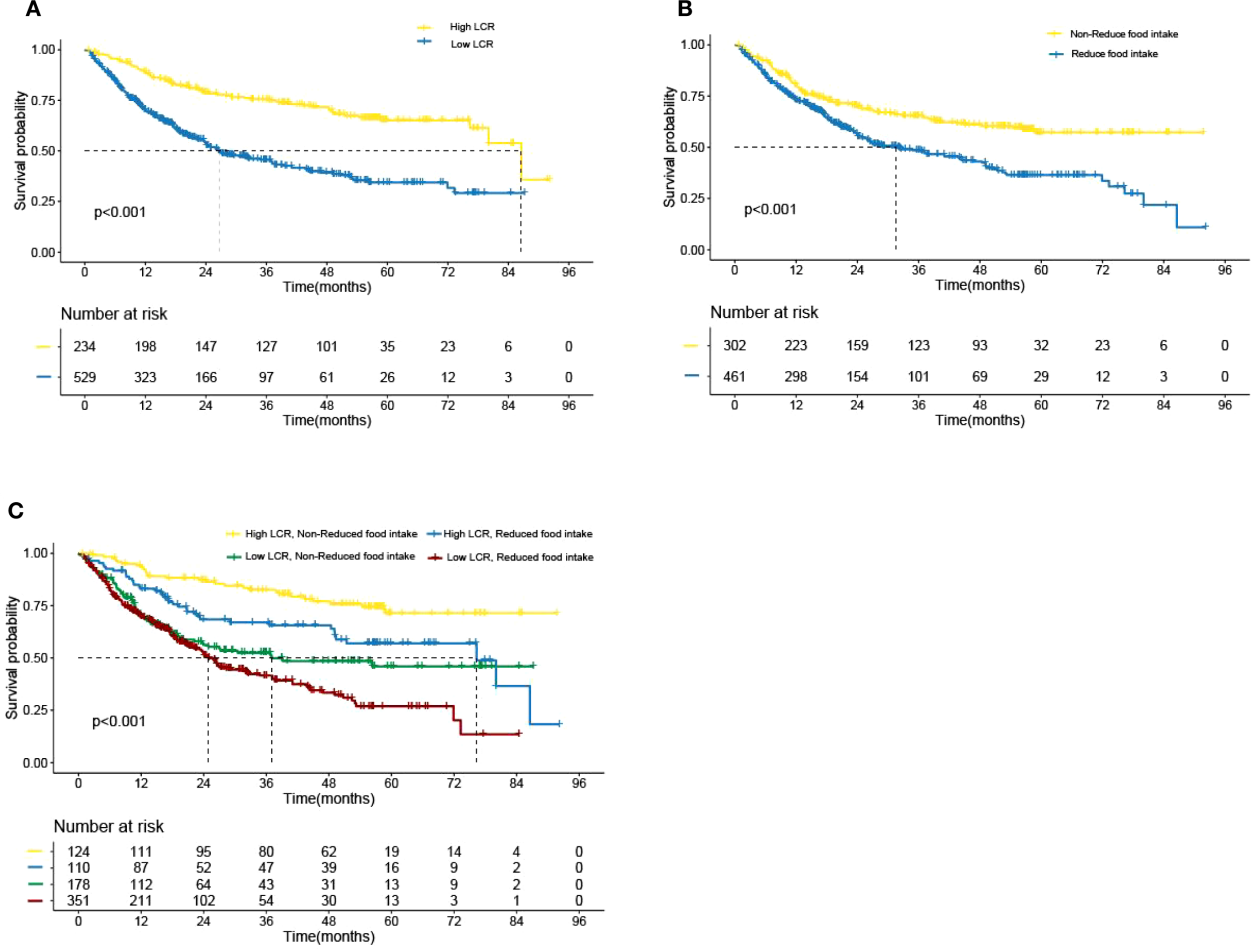
Kaplan–Meier analysis of overall survival according to the **(A)** LCR levels, **(B)** Food intake status, **(C)** LCR combined with Food intake, respectively. LCR, Lymphocyte-to-CRP ratio. .

**Figure 3:**
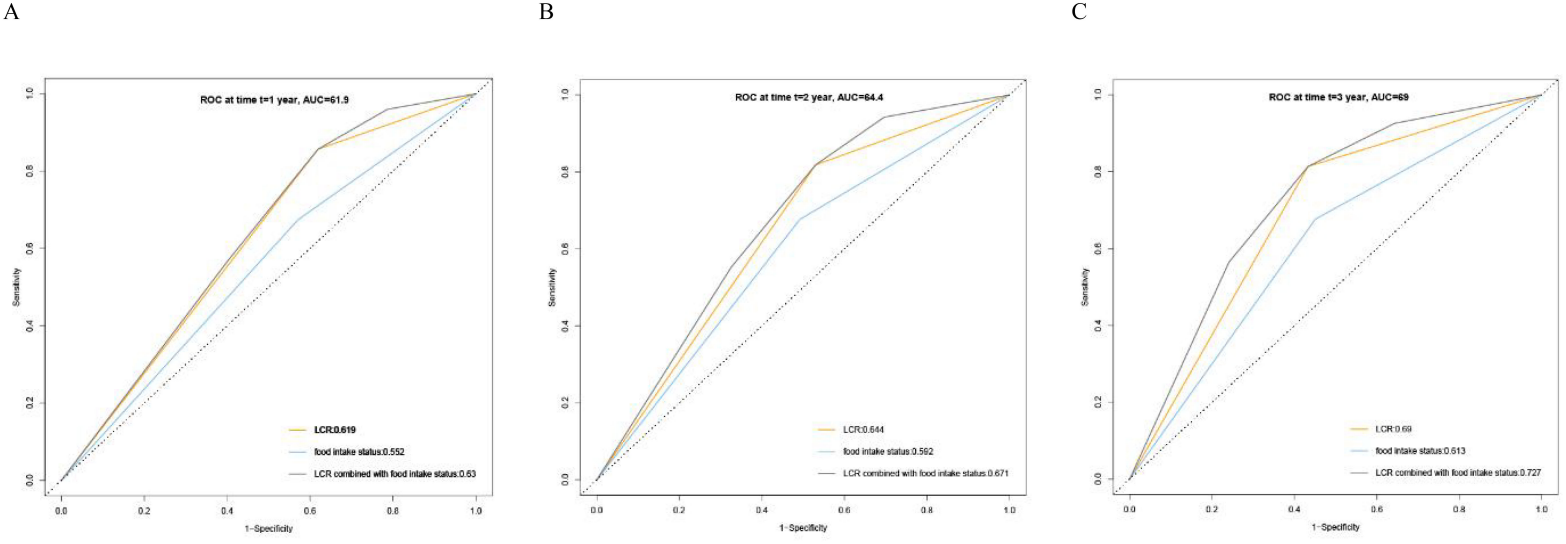
Receiver operating characteristics (ROC) curve for LCR, Food intake status and LCR combined with Food intake based on overall survival. **(A)** ROC curves for 1-year mortality, **(B)** ROC curves for 2-year mortality, **(C)** ROC curves for 3-year mortality. LCR, Lymphocyte-to-CRP ratio; AUC: area under the curve.

Kaplan–Meier curve results indicated that patients with low LCR had unfavorable survival than those patients with high LCR (**Figure 2A**). The survival time of patients without reduced food intake was longer than that of patients with reduced food intake (**Figure 2B**). In the model constructed by LCR and food intake, patients with low LCR and reduced food intake had the poorest survival compared with those of the other three groups (**Figure 2C**). When using the receiver operating characteristic (ROC) curve to evaluate the prediction effect of each model, the results showed that the AUCs of the combination of LCR and food intake in 1, 2 and 3-year were 0.63, 0.671 and 0.727, respectively (**Figures 3A-C**). These results indicated that low LCR combined with reduced food intake could be a useful indicator of OS in patients with GC.

### 3.6 Stratified analyses by potential effect modifiers

**Figure 4:**
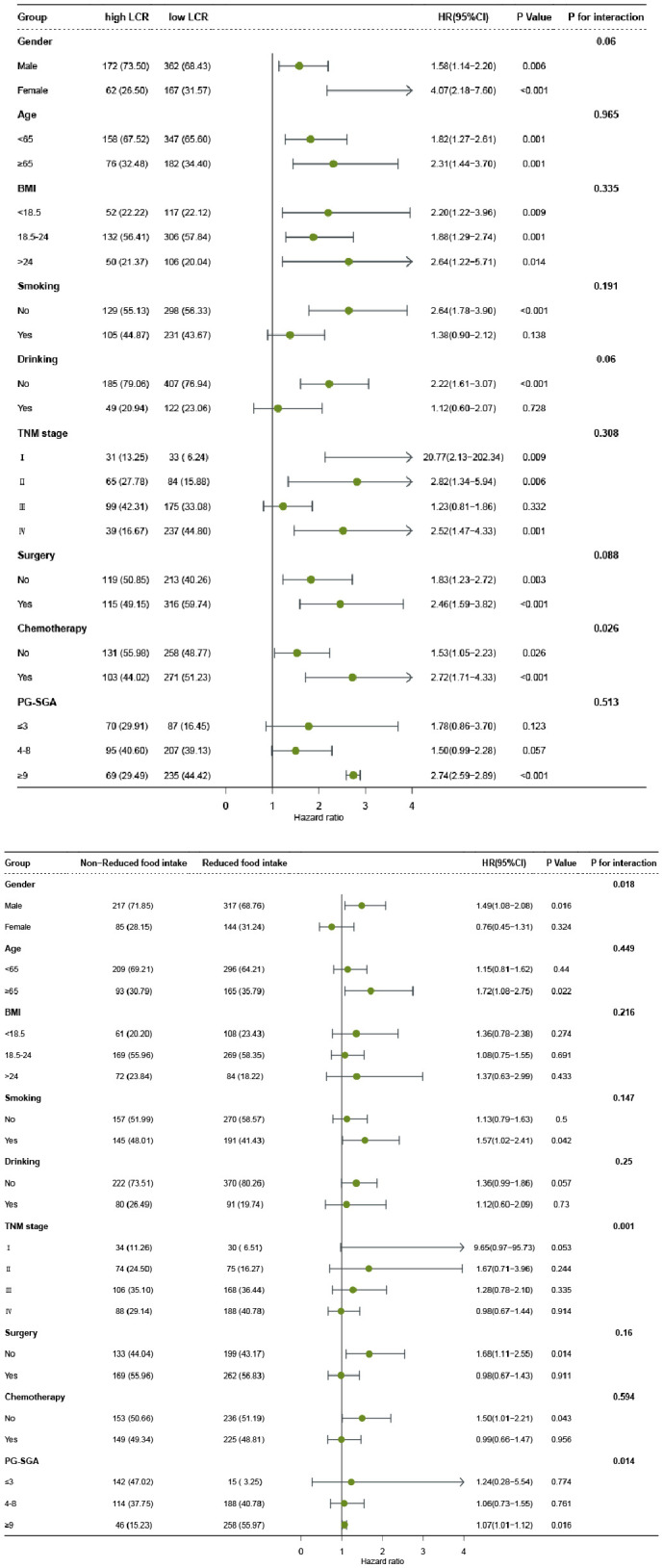
The relationship between LCR or Food intake and all-cause mortality of patients with gastric cancer in different subgroups. Notes: The cox regression model was used to calculate hazard ratios (HRs) and 95% confidence interval (CI). Each subgroup was adjusted for age, gender, smoking, drinking, tumor stage, BMI, KPS, PG-SGA, surgery, radiotherapy, chemotherapy. LCR, Lymphocyte-to-CRP ratio; BMI: body mass index; PG-SGA, Patient-Generated Subjective Global Assessment.

In order to explore the interaction of LCR or food intake with other factors in the OS of patients with GC, we performed a further interaction analysis. The association between LCR and prognosis was not significantly modified by age (*P* for interaction=0.965), BMI (*P* for interaction=0.335), smoking (*P* for interaction=0.191), TNM stage (*P* for interaction=0.308), PG-SGA (*P* for interaction=0.513), but tended to be modified by gender (*P* for interaction=0.06), drinking (*P* for interaction=0.06), surgery (*P* for interaction=0.088), and chemotherapy (*P* for interaction=0.026). Moreover, the results also revealed that food intake had an interaction with gender (*P* for interaction=0.018), TNM stage (*P* for interaction=0.001), and PG-SGA (*P* for interaction=0.014) but not with other factors (**Figure 4**). We also compared the C-indices of LCR and various indicators across different subgroups, and the results showed that LCR exhibited excellent C-indices among different genders, elderly patients, different BMI categories, and different stages (**Supplementary Table S4**). Subsequently, we conducted a combined analysis of chemotherapy and LCR. The results showed that compared with patients with high LCR who did not receive chemotherapy, the risk of death was significantly higher in patients with low LCR who received chemotherapy (HR = 2.39, 95%CI: 1.65-3.45), (**Supplementary Figure S4**). In the sensitivity analysis, we excluded 36 participants with chronic inflammatory diseases. The results showed that compared with patients with High LCR and non-reduced food intake, patients with Low LCR and reduced food intake still had the poorest prognosis (HR = 2.89, 95%CI: 1.83-4.93)(**Supplementary Table S5**).

## 4 Discussion

In this study, we verified that LCR is the best indicator for assessing the inflammation burden in GC by comparing the prediction accuracy of 16 systemic inflammatory indicators including CRP, LCR, PNI, NLR, GLR, ALI, SII, CAR, CONUT, mGPS, GNRI, mGNRI, AGR, NRI, PLR, and LCS using clinical data derived from a large cohort of patients with GC. There was a significant inverse relationship between LCR and all-cause mortality. Notably, a 21% decrease in mortality risk was observed per SD increase in LCR. The optimal cut-off point for LCR was 6451.6. Patients with reduced food intake had lower LCR than those patients without reduced food intake (*P* < 0.001). Low LCR had combined effects with reduced food intake on unfavorable OS of patients with GC. The prognostic ROC curves showed that the AUCs of the combination of LCR and food intake in 1, 2 and 3-year were 0.63, 0.671 and 0.727, respectively. Given the significant prevalence of patients with GC, our observations are likely to improve the prediction and stratification of prognosis for these patients.

Unlike the traditional predictive models constructed based on clinical features, we incorporated more laboratory indicators and adjusted for clinically relevant features (18). Some studies have compared the validity of several systemic inflammatory indicators in predicting the prognosis of malignancies. In a single-center retrospective study, patients with unresectable or recurrent gastric cancer who had a low LCR before first-line and second-line chemotherapy had a significantly worse prognosis than those with a high LCR. Nutritional intervention during chemotherapy induction may lead to a better prognosis (19). Similar to our study, improvements in nutrition and food intake and an increase in LCR exert a synergistic effect. Suzuki et al. compared the prognostic value of 16 systemic inflammatory biomarkers and found LCR had the highest accuracy to predict OS and was the only biomarker that was an independent predictor of both OS and disease-free survival, however, that study was focused on patients with stage II or III colon cancer (20).A recent study on gastric cancer also explored the predictive value of 18 preoperative immune, inflammatory, and nutritional biomarkers and their optimal cut-off values for OS and disease-free survival (DFS) in patients with gastric adenocarcinoma who underwent gastrectomy. The results showed that the NLR, monocyte systemic inflammation index, and PNI are the most promising preoperative biomarkers for predicting patients’ OS and DFS (21). However, the study did not include LCR as an indicator. In our analysis, 16 systemic inflammatory indicators were compared, and the result indicated that LCR was the best indicator in assessing OS in patients with GC. Although there were differences in cancer type for prognostic scores among these studies, all of them suggest that LCR is a significant predictive biomarker common to above studies. Therefore, among the various systemic inflammatory indicators, LCR may be the most reliable biomarker to predict the prognosis of gastrointestinal tumors.

The value of LCR is determined by only two key serum markers: serum CRP concentration and total lymphocyte count. Serum CRP is the most representative clinical marker of acute systemic inflammation, which is mainly produced by liver cells. The rapid increase in serum CRP concentration is related to pro-inflammatory factors, such as interleukin-1 (IL-1), IL-6, IL-8, and tumor necrosis factor α. Such factors are upregulated during inflammatory response in the body and promote the progression and metastasis of malignant tumors by accelerating angiogenesis (22, 23). A decrease in the number of lymphocytes can be a factor that deteriorates the values of LCR. Pro-inflammatory cytokines can mediate the recruitment of circulating myeloid cells to the tumor, and CD8+ T cells are decreased due to direct or indirect immunosuppression by intratumor myeloid cells (24). Thus, lymphopenia reflects the presence of immunosuppression, which promotes cancer progression. Previous research by Clark et al. showed that a low pre-operative lymphocyte level rather than NLR is a good predictor of poor prognosis for pancreatic ductal adenocarcinoma (25). The increase in circulating lymphocytes and the decrease in serum CRP levels may reflect the good health status of patients (5). Consistently, the findings in our analysis showed that there was a significant inverse relationship between LCR and all-cause mortality in patients with GC.

Low LCR may indicate a status of immune escape, and systemic inflammatory response, which exhausts nutrition and energy in patients with cancer and may increase the risk of malnutrition in more than half of the patients. Moreover, reduced food intake occurs in most patients with GC as a result of the disease itself and mechanical factors (26, 27). Studies have confirmed the potential correlation between reasonable food intake and better therapeutic responses to targeted or immune therapy in patients with GC (28, 29). The present study demonstrated that LCR levels were lower in patients with reduced food intake than in those without reduced food intake. When combining LCR and food intake, we found that patients with low LCR and reduced food intake had the worst prognosis. The underlying mechanism of this association can be further explained by the interactive effect of the “inflammation-malnutrition-immune function” axis. In a state of chronic inflammation, pro-inflammatory factors (such as IL-6 and TNF-α) related to tumors or diseases can regulate the appetite center through specific molecular pathways. Studies have shown that IL-6 can activate the hypothalamic JAK/STAT3 signaling pathway, upregulate the expression of appetite-suppressing factors (e.g., pro-opiomelanocortin, POMC), and simultaneously inhibit the release of appetite-promoting factors (e.g., neuropeptide Y, NPY), thereby directly suppressing the activity of the appetite center and leading to a decrease in patients’ voluntary food intake (30, 31). In turn, insufficient food intake impairs lymphocyte function through nutritional deficiency: a lack of proteins, essential fatty acids, and vitamins inhibits the proliferation, differentiation of lymphocytes, and their ability to secrete cytokines (such as IFN-γ and IL-2), resulting in a reduction in the peripheral blood lymphocyte count. Meanwhile, malnutrition further exacerbates the persistence of inflammatory responses, thereby forming a vicious cycle (32, 33). Ultimately, these factors collectively lead to a decrease in LCR levels. This mechanism also explains why the combined assessment of food intake status and LCR can more accurately reflect the overall state of patients: food intake directly reflects nutritional reserve, while LCR reflects the immune-inflammatory balance. The association between the two essentially represents the coordinated changes in the “nutrition-immune-inflammation” system. Therefore, reduced inflammation and enhanced nutritional support may contribute to tumorigenesis prevention and improve long-term outcomes in patients with poor inflammation-nutrition-based prognostic scores.

This study found a significant interaction between LCR and chemotherapy. This finding suggests that chemotherapy does not merely affect prognosis as an independent treatment factor; instead, it reshapes the association pattern between LCR and patient prognosis by regulating the immune-inflammatory balance system represented by LCR. Mechanistically, on one hand, for patients with good baseline immune reserve, chemotherapy regimens with immunogenic cell death effects (e.g., those containing anthracyclines or platinum agents) can induce tumor cells to release antigens and damage-associated molecular patterns (34). This activates dendritic cell-mediated antigen presentation, promotes the proliferation and activation of CD8+ cytotoxic T cells and NK cells, and increases the immune weight in LCR, ultimately strengthening the positive association between high LCR and longer OS (35). On the other hand, in patients receiving high-intensity chemotherapy (e.g., dose-dense alkylating agent regimens) or with baseline chronic inflammation, chemotherapy may cause excessive lymphocyte apoptosis and exhaustion, or induce acute inflammatory responses due to normal tissue damage (36). This leads to a sharp increase in CRP levels and a higher proportion of the “inflammatory component” in LCR, resulting in a significant weakening of the prognostic advantage of high LCR, or even a shift in the direction of the association.

The present study was innovative in the following ways. For the first time, we used the multi-center data and confirmed that LCR is the best indicator for assessing the inflammation burden by comparing the prediction accuracy of 16 systemic inflammatory indicators, representing the GC study with inclusion of the highest number of indicators. Moreover, the level of evidence-based medicine of multi-center study is theoretically superior to that of single-center study, which makes the results more universal and applicable (37). Finally, Eastern and Western gastric cancer patients exhibit significant differences in epidemiological characteristics, clinicopathological characteristics, tumor biology, treatment modalities, and drug selection (38). Advanced GC accounts for more than 80% of cases in China in contrast to early GC, which accounts for 60% of cases in Japan and South Korea (39). Meanwhile, this study focuses on the Asian population and provides more insights into the characteristics of gastric cancer in Asia. In our study, 76.8% and 72.1% of the patients were advanced GC according to the classification of LCR and food intake, respectively. Samples from different populations are needed to assess the role of the combination of LCR and food intake in the prognosis of patients with GC.

The present study had some limitations. First, we only conducted a number of retrospective correlational analyses, and thus cannot establish a causal relationship. Second, we only used data from China, and large samples of western data are still needed to further validate the findings. In addition, there is a lack of basic research exploring the specific mechanism by which LCR and food intake affect the oncological efficacy in patients with GC. Thirdly, we failed to include detailed treatment modalities for gastric cancer patients, such as chemotherapy regimens and surgical approaches. This may introduce unavoidable bias, as the line of regimens and differences in surgical procedures can seriously affect patients’ prognosis. Fourthly, monocytes play an important role in the inflammatory process of carcinogenesis; however, due to issues related to data quality control and data missing, we did not include the relevant indicators. However, the completeness of the clinicopathological data and the relatively large sample size may partially compensate for this limitation.

In conclusion, LCR was the best indicator for assessing the inflammation burden and was inversely correlated with patients’ all-cause mortality. LCR levels were lower in patients with reduced food intake than in those without reduced food intake. Combined assessment of LCR and food intake contributes to prognostic stratification of GC. It is important to focus on the baseline LCR and food intake and take active therapeutic measures to reduce inflammation and increase nutrition to improve outcomes of affected patients.

## Data Availability

The data analyzed in this study is subject to the following licenses/restrictions: Additional data related to this study is available upon request to authors/corresponding author. Requests to access these datasets should be directed to shihp@ccmu.edu.cn.
