# Supplementary material for: Combined assessment of inflammation and food intake contributes to prognostic stratification of gastric cancer
Source: Front Oncol. 2025 Oct 23;15:1669838. doi: 10.3389/fonc.2025.1669838 (PMC12588835; doi:10.3389/fonc.2025.1669838)
Supplement: Supplementary file 1 [file Table1.docx]

**Supplementary Methods**

**Food Assessment Scale**

In the past month, compared with my usual eating situation: No change (Non-Reduced food intake); More than usual (Non-Reduced food intake); Less than usual (Reduced food intake).

**Table S1.** Calculation methods of systemic inflammatory indicators.

| Indicators | Score | Calculation formulas |
| --- | --- | --- |
| LCR |  | 10000× lymphocytes (10^9/L)/CRP (mg/L) |
| PNI |  | Serum albumin concentration (g/L) + 5× absolute lymphocyte count(10^9/L) |
| CAR |  | CRP (mg/L)/albumin(g/L) |
| ALI |  | 0.1× BMI× albumin(g/L)/NLR |
| mGNRI |  | 14.89/CRP (mg/L) + 41.7 × [present body weight (kg)/ideal body weight(kg)] |
| NRI |  | 1.519×serum albumin concentration(g/L) + 41.7× [present body weight (kg)/ideal body weight(kg)] |
| GNRI |  | 1.489 × serum albumin(g/L) + 41.7 × [present body weight (kg)/ideal body weight(kg)] |
| AGR |  | Albumin/globulin Ratio |
| NLR |  | Nuetrophil/lymphocyte Ratio |
| GLR |  | Glucose (mmol/L)/ lymphocyte Ratio |
| PLR |  | Platelet/lymphocyte Ratio |
| SII |  | Nuetrophil× platelet/lymphocyte Ratio |
| LCS | 0 | Lymphocyte count≥10^9/L and CRP≤3mg/L |
|  | 0 | Lymphocyte count<10^9/L and CRP≤3mg/L |
|  | 1 | Lymphocyte count≥10^9/L and CRP>3mg/L |
|  | 2 | Lymphocyte count<10^9/L and CRP>3mg/L |
| CONUT | 0-1 | Albumin(g/dL): >3.50(0), 3.00-3.49(2), 2.50-2.99(4), <2.50(6)  Total lymphocyte count(/ml): >1600(0), 1200-1599(1), 800-1199(2), <800(3)  Total cholesterol(mg/dL): >180(0), 140-180(1), 100-139(2), <100(3) |
|  | 2-4 |  |
|  | 5-12 |  |
| mGPS | 0 | CRP≤10mg/L and albumin≥35g/L |
|  | 0 | CRP≤10mg/L and albumin<35g/L |
|  | 1 | CRP>10mg/L |
|  | 2 | CRP>10mg/L and albumin<35g/L |

LCR, Lymphocyte-to-CRP ratio; PNI, Prognostic nutritional index; NLR, Neutrophil-to-lymphocyte ratio; GLR, Glucose-to-lymphocyte ratio; ALI, Advanced lung cancer inflammation index; SII, Systemic immune inflammation index; CAR, C-reactive protein-to-albumin ratio; CONUT score, Controlling nutritional status score; mGPS, modified Glasgow prognostic score; GNRI, Geriatric nutritional risk index; mGNRI, modified Geriatric nutritional risk index; AGR, Albumin-to-globulin ratio; NRI, Nutritional risk index; PLR, Platelet-to-lymphocyte ratio; LCS, Lymphocyte-to-CRP ratio score.

**Table S2.** Detailed baseline characteristics of patients with gastric cancer.

| **Characteristics** | **Overall Patients (n=763)** |
| --- | --- |
| Population Characteristic |  |
| Gender, male, n (%) | 534 (70.0%) |
| Age, years, mean (SD) | 59.12 (11.87) |
| BMI, kg/m^2^, mean (SD) | 21.18 (3.14) |
| Clinical Characteristic |  |
| TNM stage, n (%) |  |
| I | 64 (8.4%) |
| II | 149 (19.5%) |
| III | 274 (35.9%) |
| IV | 276 (36.2%) |
| surgery, yes, n (%) | 431 (56.5%) |
| Radiotherapy, yes, n (%) | 10 (1.3%) |
| Chemotherapy, yes, n (%) | 374 (49.0%) |
| Albumin, g/L, mean (SD) | 38.73 (5.38) |
| Hemoglobin, g/L, mean (SD) | 115.34 (31.02) |
| CRP, mg/L, mean (SD) | 15.20 (29.65) |
| NLR, mean (SD) | 3.41 (3.76) |
| PLR, mean (SD) | 174.41 (107.38) |
| GLR, mean (SD) | 4.50 (3.59) |
| ALI, mean (SD) | 42.23 (30.49) |
| SII, mean (SD) | 833.77 (1351.50) |
| CAR, mean (SD) | 0.44 (0.94) |
| CONUT score, n (%) |  |
| 0-1 | 289 (37.9%) |
| 2-4 | 353 (46.3%) |
| 5-12 | 121 (15.9%) |
| mGPS, n (%) |  |
| 0 | 558 (73.1%) |
| 1 | 113 (14.8%) |
| 2 | 92 (12.1%) |
| GNRI, mean (SD) | 95.76 (9.31) |
| mGNRI, mean (SD) | 46.77 (16.36) |
| AGR, mean (SD) | 1.41 (0.33) |
| PNI, mean (SD) | 46.38 (6.76) |
| NRI, mean (SD) | 96.92 (9.46) |
| LCS, n (%) |  |
| 0 | 201 (26.3%) |
| 1 | 464 (60.8%) |
| 2 | 98 (12.8%) |
| LCR, mean (SD) | 9338.14 (18377.67) |
| KPS, mean (SD) | 84.43 (11.32) |
| PG-SGA, mean (SD) | 7.75 (4.74) |
| Reduced food intake, yes, n (%) | 461 (60.4) |

BMI, body mass index; CRP, C-reactive protein; LCR, Lymphocyte-to-CRP ratio; PNI, Prognostic nutritional index; NLR, Neutrophil-to-lymphocyte ratio; GLR, Glucose-to-lymphocyte ratio; ALI, Advanced lung cancer inflammation index; SII, Systemic immune inflammation index; CAR, C-reactive protein-to-albumin ratio; CONUT score, Controlling nutritional status score; mGPS, modified Glasgow prognostic score; GNRI, Geriatric nutritional risk index; mGNRI, modified Geriatric nutritional risk index; AGR, Albumin-to-globulin ratio; NRI, Nutritional risk index; PLR, Platelet-to-lymphocyte ratio; LCS, Lymphocyte-to-CRP ratio score; KPS, Karnofsky Performance Status; PG-SGA, Patient-Generated Subjective Global Assessment.

**Table S3.** The *C*-index of systemic inflammatory indicators for OS in patients with gastric cancer.

| Indicators | *C*-index(95%CI) |
| --- | --- |
| LCR | 0.642(0.610, 0.674) |
| PNI | 0.632(0.601, 0.664) |
| CAR | 0.630(0.598, 0.662) |
| CRP | 0.626(0.594, 0.658) |
| ALI | 0.622(0.591, 0.653) |
| mGNRI | 0.621(0.589, 0.652) |
| LCS | 0.616(0.588, 0.645) |
| NRI | 0.610(0.577, 0.643) |
| GNRI | 0.610(0.577, 0.643) |
| AGR | 0.608(0.577, 0.640) |
| NLR | 0.607(0.575, 0.639) |
| CONUT | 0.606(0.572, 0.639) |
| GLR | 0.598(0.565, 0.632) |
| PLR | 0.598(0.566, 0.630) |
| SII | 0.596(0.564, 0.629) |
| mGPS | 0.568(0.541, 0.596) |

LCR, Lymphocyte-to-CRP ratio; PNI, Prognostic nutritional index; NLR, Neutrophil-to-lymphocyte ratio; GLR, Glucose-to-lymphocyte ratio; ALI, Advanced lung cancer inflammation index; SII, Systemic immune inflammation index; CAR, C-reactive protein-to-albumin ratio; CONUT score, Controlling nutritional status score; mGPS, modified Glasgow prognostic score; GNRI, Geriatric nutritional risk index; mGNRI, modified Geriatric nutritional risk index; AGR, Albumin-to-globulin ratio; NRI, Nutritional risk index; PLR, Platelet-to-lymphocyte ratio; LCS, Lymphocyte-to-CRP ratio score; CRP, C-reactive protein; OS, overall survival.

**Table S4.** The *C*-index of systemic inflammatory indicators for OS in patients with gastric cancer indifferent subgroup.

| Indicators | Men | Women | <65y | ≥65y | BMI<18.5 | BMI:18.5-24 | BMI≥24 | TNM: I,II | TNM: III,IV |
| --- | --- | --- | --- | --- | --- | --- | --- | --- | --- |
| LCR | 0.665 | 0.628 | 0.636 | 0.695 | 0.630 | 0.665 | 0.632 | 0.633 | 0.657 |
| PNI | 0.642 | 0.617 | 0.649 | 0.616 | 0.623 | 0.642 | 0.630 | 0.630 | 0.632 |
| CAR | 0.635 | 0.601 | 0.632 | 0.611 | 0.619 | 0.632 | 0.628 | 0.621 | 0.636 |
| CRP | 0.517 | 0.599 | 0.567 | 0.662 | 0.628 | 0.641 | 0.611 | 0.622 | 0.628 |
| ALI | 0.631 | 0.601 | 0.630 | 0.606 | 0.625 | 0.626 | 0.611 | 0.620 | 0.626 |
| mGNRI | 0.633 | 0.603 | 0.628 | 0.613 | 0.609 | 0.621 | 0.630 | 0.612 | 0.628 |
| LCS | 0.621 | 0.600 | 0.620 | 0.600 | 0.626 | 0.630 | 0.601 | 0.622 | 0.617 |
| NRI | 0.623 | 0.589 | 0.626 | 0.599 | 0.604 | 0.632 | 0.622 | 0.630 | 0.634 |
| GNRI | 0.633 | 0.593 | 0.597 | 0.622 | 0.600 | 0.611 | 0.612 | 0.605 | 0.613 |
| AGR | 0.624 | 0.587 | 0.611 | 0.598 | 0.593 | 0.609 | 0.606 | 0.607 | 0.618 |
| NLR | 0.613 | 0.586 | 0.591 | 0.611 | 0.605 | 0.610 | 0.606 | 0.622 | 0.606 |
| CONUT | 0.591 | 0.613 | 0.606 | 0.603 | 0.611 | 0.612 | 0.599 | 0.598 | 0.615 |
| GLR | 0.549 | 0.618 | 0.577 | 0.603 | 0.593 | 0.595 | 0.599 | 0.601 | 0.592 |
| PLR | 0.595 | 0.599 | 0.603 | 0.589 | 0.599 | 0.595 | 0.602 | 0.586 | 0.586 |
| SII | 0.612 | 0.531 | 0.606 | 0.581 | 0.594 | 0.588 | 0.595 | 0.599 | 0.594 |
| mGPS | 0.555 | 0.582 | 0.549 | 0.587 | 0.566 | 0.578 | 0.567 | 0.577 | 0.568 |

Table S5 Sensitivity analysis

|  | Adjusted model | |
| --- | --- | --- |
|  | HR 95%CI | *p*-value |
| High LCR, Non-Reduced food intake | ref |  |
| High LCR, Reduced food intake | 1.70 (1.02,2.26) | 0.043 |
| Low LCR, Non-Reduced food intake | 2.33 (1.21,3.63) | <0.001 |
| Low LCR, Reduced food intake | 2.89 (1.83,4.93) | <0.001 |
| P for trend |  | <0.001 |

Adjusted model was adjusted for age, gender, smoking, drinking, tumor stage, BMI, KPS, PG-SGA, surgery, radiotherapy, chemotherapy..


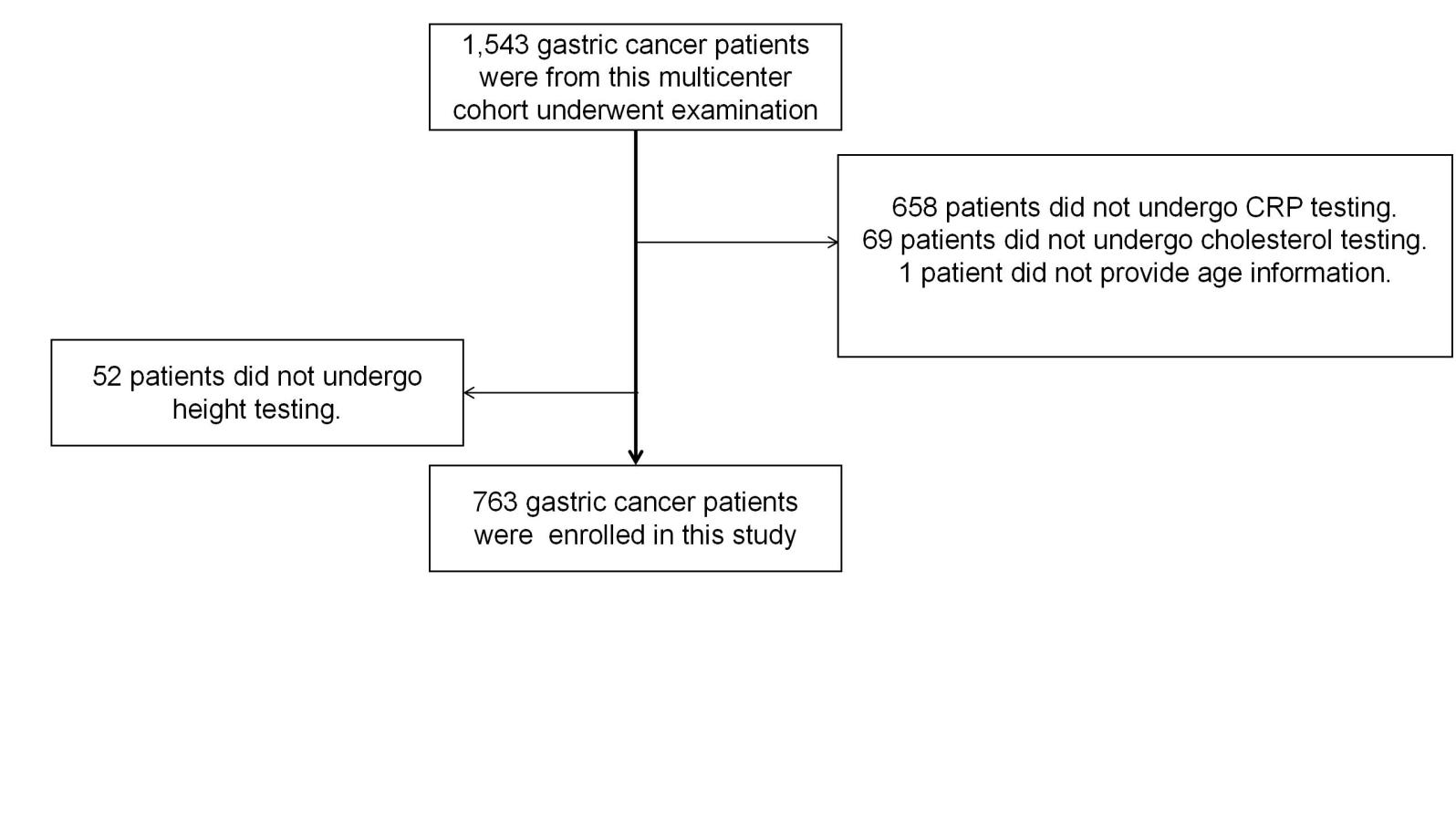


**Fig. S1.** The flow chart.


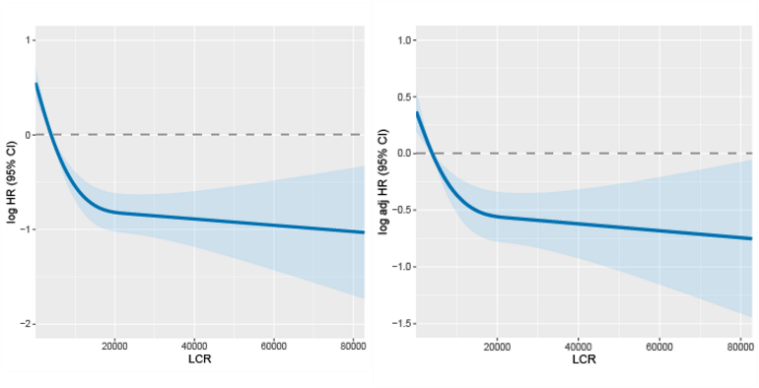


**Fig. S2.** The association between LCR and all-cause mortality in patients with gastric cancer. The spline was adjusted for cofounders including age, gender, smoking, drinking, TNM stage, BMI, KPS, PG-SGA, surgery, radiotherapy, chemotherapy. LCR, Lymphocyte-to-CRP ratio.


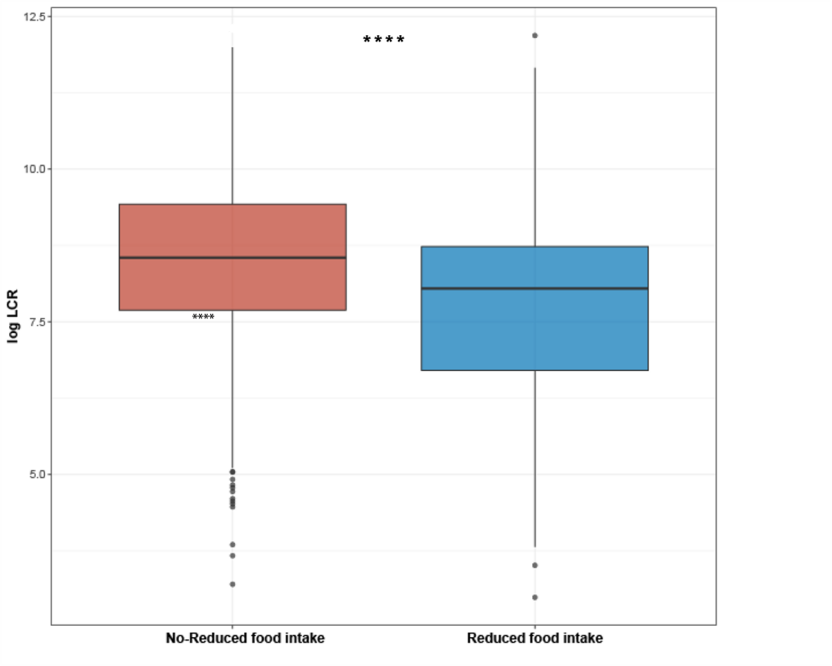


**Fig. S3.** The distribution of LCR (log transformation) in Reduced food intake and Non-Reduced food intake groups. LCR, Lymphocyte-to-CRP ratio. **** p-value<0.001.


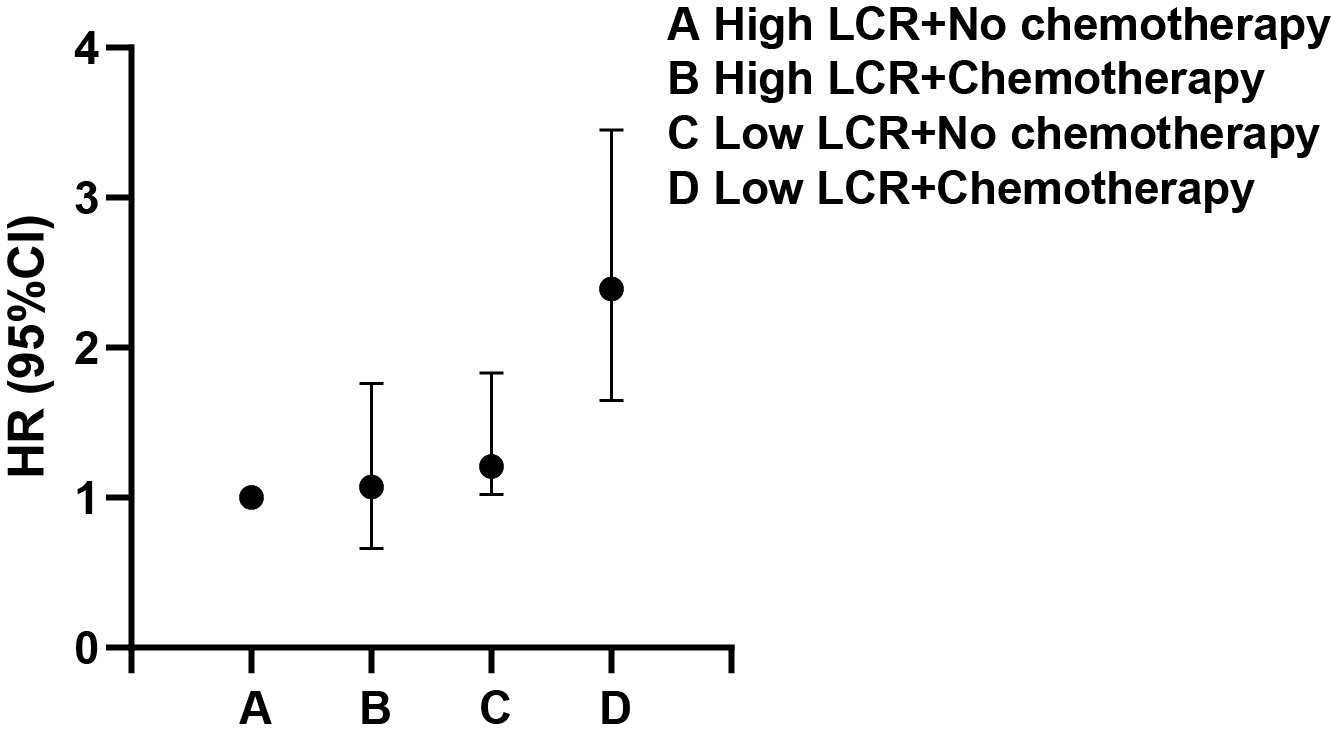


**Fig. S4. The Relationship Between Combined Analysis of Chemotherapy and LCR and Prognosis**
